# Kinetic modeling and process analysis for photo-production of β-carotene in *Dunaliella salina*

**DOI:** 10.1186/s40643-022-00495-6

**Published:** 2022-01-17

**Authors:** Yimei Xi, Jiali Zhang, Fantao Kong, Jian Che, Zhanyou Chi

**Affiliations:** 1 Key Laboratory of Industrial Ecology and Environmental Engineering (Ministry of Education, China), School of Environmental Science and Technology, Dalian University of Technology, Dalian, 116024 China; 2 School of Mathematical Sciences, Dalian University of Technology, Dalian, 116024 China; 3 School of Bioengineering, Dalian University of Technology, Dalian, 116024 China; 4 Dalian Xinyulong Marine Biological Seed Technology Co. Ltd, Dalian, 116200 China

**Keywords:** *Dunaliella salina*, Dynamic kinetic modeling, Cultivation optimization, Environmental factors, β-Carotene production

## Abstract

*Dunaliella salina* is a green microalga with the great potential to generate natural β-carotene. However, the corresponding mathematical models to guide optimized production of β-carotene in *Dunaliella salina* (*D. salina*) are not yet available. In this study, dynamic models were proposed to simulate effects of environmental factors on cell growth and β-carotene production in *D. salina* using online monitoring system. Moreover, the identification model of the parameter variables was established, and an adaptive particle swarm optimization algorithm based on parameter sensitivity analysis was constructed to solve the premature problem of particle swarm algorithm. The proposed kinetic model is characterized by high accuracy and predictability through experimental verification, which indicates its competence for future process design, control, and optimization. Based on the model established in this study, the optimal environmental factors for both β-carotene production and microalgae growth were identified. The approaches created are potentially useful for microalga *Dunaliella salina* cultivation and high-value β-carotene production.

**Graphical Abstract:**

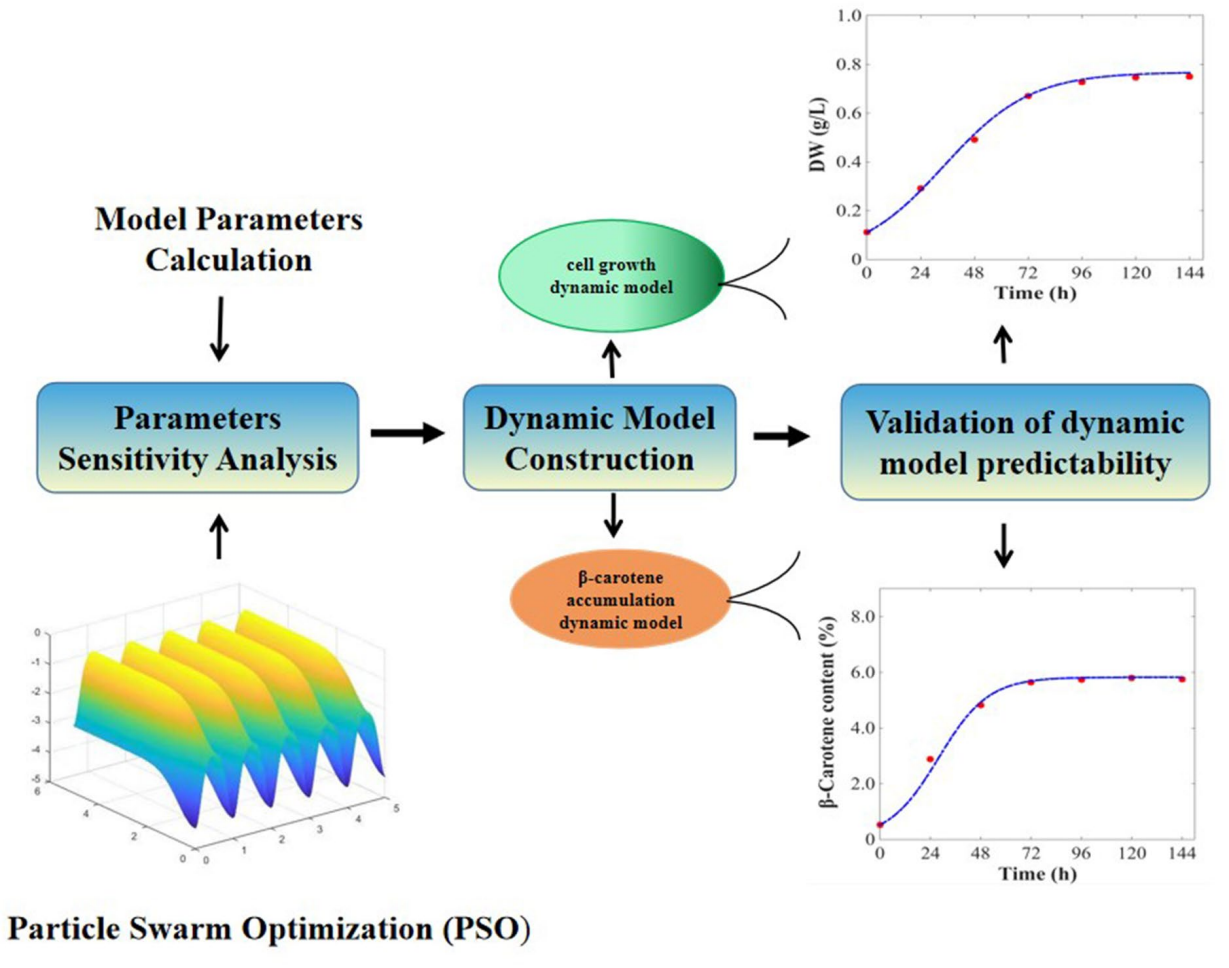

**Supplementary Information:**

The online version contains supplementary material available at 10.1186/s40643-022-00495-6.

## Introduction

In the last decades, massive investments were done on microalgae industry, mainly due to their capacity to synthesize lipids for biofuel production or synthesize the carotenoid for high-value product production (Chew 2017; Kong et al. 2018; Salome and Merchant 2019). β-Carotene is a high-valued carotenoid pigment with wide applications in the cosmetic, pharmaceutical, and food industries (Coppens et al. 2016; Gateau et al. 2017; Paillie-Jimenez et al. 2020). However, the supply of natural β-carotene still falls short of demand at present (Henriquez et al. 2016). The green microalga *Dunaliella salina* has been regarded as one of the best candidates for β-carotene production due to its high β-carotene content (up to 10%) (Benamotz et al. 1982; Xi et al. 2020).

Microalgae require macronutrients and micronutrients for photosynthesis, which are important for growth and product accumulation. The optimal temperature and light are also critical for rapid metabolism and biomass productivity in microalgae (Viruela et al. 2021; del Rio-Chanona et al. 2017). During the past years, many studies have been carried out to evaluate the optimal operating conditions for β-carotene production in *Dunaliella* sp. For example, high irradiance, high temperature, oxidative stress, and nitrogen-deprivation have been found to significantly stimulate the accumulation of β-carotene (Fachet et al. 2016; Wu et al. 2016; Kim et al. 2013). Although there are extensive indoor measurements for the microalgae system (Zhu et al. 2021, 2018b), much less effort has been focused on the modeling of these stages to determine the optimal operating conditions. To optimize reactor design and predict its performance, understanding of simultaneous effects of different environmental and operational variables on microalgae culture is necessary. Mathematical models can be used to study the effects of the environmental and operational variables, which are related to the output variables (e.g., biomass productivity and bioproduct production), allowing the effect of changing the input variables to be studied without the need for individual experimental tests (Viruela et al. 2021; del Rio-Chanona et al. 2018). To successfully conduct process control and make optimization, it is essential to construct a highly accurate kinetic model, which can be capable of well predicting the dynamic behavior of the underlying biosystem. Meanwhile, model-based process design is also considered to be one of the most effective tools to accomplish the transfer of bioprocess from laboratory short-term scale to industrial long-term scale (Viruela et al. 2021; del Rio-Chanona et al. 2017). Despite its importance, model-based process design for β-carotene production still remains to be elucidated.

Previous modeling studies have taken on the specific challenge of modeling growth in microalgae systems (Fachet et al. 2014; Viruela et al. 2021). Although some dynamic models have been constructed to simulate this process. However, most of the environmental parameters have not been considered in biokinetics in those models, which limited their application. For example, a photobioreactor model that deals only with both light and nitrogen limitation has been proposed through Beer–Lamberts Law and the Droop Equation, respectively (Bernard et al. 2009). Microalgae growth is reported to be related to light intensity and the intracellular nitrogen concentration or quota, but the effect of other relevant parameters such as temperature or inorganic carbon concentration which restrict the applicability of microalgae has not been included. Mathematical models have been used to predict and optimize the microalgae biomass and astaxanthin production, and specific variables including light intensity, temperature, retention time, and nutrients’ concentration have been used to monitor the process performance and construct models (Zhang et al. 2015, 2016). However, due to the specificity between microalgae species and the induction stage of carotenoid, where cells stop growing and carotenoid commences to accumulate are very difficult to model.

Droop, Monod, and Andrew models have been extensively applied to predict biomass growth rate under a single substrate or nutrient condition, such as phosphorus (del Rio-Chanona et al. 2017), nitrogen (del Rio-Chanona et al. 2017; Liu et al. 2018), carbon (Straka and Rittmann 2019), and light (Holdmann et al. 2018; Zhang et al. 2015). Previous models are able to accurately estimate biomass productivity when the temperature is within a range of values enabling microalgae growth (Zhang et al. 2016). Integrated experimental–computational frameworks that have the ability to predict biomass growth and product accumulation under different growing conditions, which will help to optimize the process performance, operating conditions, and scale-up of cultivation systems for commercialization and industrial applicability (Zeriouh et al. 2017).

Nevertheless, kinetic modeling of simultaneous co-limitation of growth media elements (e.g., nitrogen and carbon) and environmental factors (e.g., light and temperature) has not been reported yet. Additionally, while carotenoid accumulation has been considered to be proportional to biomass growth, the effects of abiotic stress toward enhancement of carotenoid productivity have been recently shown through a new kinetic model considering biomass growth and carotenoid accumulation as two different dynamic variables (Zhang et al. 2016). Dynamic simulation is an effective tool to determine the optimal operating conditions for both laboratory-scale and industrial-scale carotenoid production processes. However, the previous models are only able to accurately estimate biomass productivity when the temperature is within a range of values enabling microalgae growth (Jiang et al. 2015). Moreover, the previous models can only predict biomass and bio-compounds production when the incident light intensity is within a range of constant, but the average light intensity received by the cells in the photobioreactors (PBRs) is underestimated (Lamers et al. 2010).

To accurately simulate the dynamic process of the β-carotene induction stage, the current study aims to construct rigorous models including the effects of temperature, average light intensity, carbon and nitrogen source on microalgal growth, and β-carotene accumulation, which to the best of our knowledge has not been reported at present. Furthermore, a sensitivity analysis was conducted to simulate various parameters on microalgae production process. The relationship between β-carotene accumulation and algal growth has also been comprehensively studied in this study, which are potentially useful for microalga *Dunaliella salina* cultivation and high-value β-carotene production.

## Material and modeling methodology

### Microalgal strain and its preculture conditions

The microalga *Dunaliella salina* (*D. salina*) CCAP 19/18 was purchased from Culture Collection of Algae and Protozoa (Windermere, United Kingdom). The strain was maintained in the medium of optimized Artificial Sea Water (ASW), composing of 1.5 M NaCl, 5 mM KNO_3_, 0.45 mM MgCl_2_·6H_2_O, 0.05 mM MgSO_4_·7H_2_O, 0.3 mM CaCl_2_·2H_2_O, 0.13 mM K_2_HPO_4_, 0.02 mM FeCl_3_, 0.02 mM EDTA, 1 mL of trace elements stock per liter with 50 mM H_3_BO_3_, 10 mM MnCl_2_·4H_2_O, 0.8 mM ZnSO_4_·7H_2_O, 0.8 mM CuSO_4_·5H_2_O, 2 mM NaMoO_4_·2H_2_O, 1.5 mM NaVO_3_, and 0.2 mM CoCl_2_·6H_2_O, and the pH was adjusted to 7.5 by addition of Tris-buffer (40 mM). *D. salina* was precultured in 500 mL conical flasks at 50 µmol·photons·m^−2^·s^−1^ light intensity and under 14 h/10 h light/dark cycles.

### Operation of photobioreactor

The microalgal cells at logarithmic phase were inoculated into a multi-device-equipped flat plate photobioreactor (also known as Algal Station) (Additional file 1: Fig. S1), which can guarantee accurate and stable light condition control as we previously described (Cao et al., 2019). In the platform of Algal Station, the cultivation temperature was automatically controlled at 25 ℃, the pH was maintained at 7.5 by computer-controlled micro-addition of CO_2_ in the bubbling air, and the cultures were agitated at 400 mL^.^min^−1^ with filtered air (0.2 μm porosity membrane). The culturing broth was sampled daily for analysis of dry weight and β-carotene content. The incident photon flux density and transmitted photon flux were recorded online at 20 min intervals*.* Each treatment was independently repeated three times.

## Analytical methods

### Growth analyses

Cell density was determined spectrophotometrically using a UV/Vis spectrophotometer (Jasco V-530, Japan) at 680 nm. The microalgal dry weight (DW) was determined according to the method we previously described (Cao et al. 2019). Briefly, with pre-weighed Whatman GF/C filters, 10 mL culture broth was filtered and washed three times with 2 mL of 0.5 M ammonium bicarbonate, and then dried below 60 °C for over 16 h until the weight was constant. The DW of the microalgae cells was calculated, according to the difference between final and initial filter weights and volume of the filtered sample.

The microalgal growth rate (*µ*_*i*_, h^−1^) was calculated by Eq. (1).

1 $$\mu_{i} = \frac{{LnDW_{i} - LnDW_{i - 1} }}{{t_{i} - t_{i - 1} }},$$

where *DW*_*i*_ and $DW_{i - 1}$ (g·L^−1^) are the biomass concentration measured at time *t*_*i*_ and *t*_*i-1*_, respectively. *t*_*i*_ and *t*_*i-1*_ are hour i and i-1 when the culture broth was sampled.

### β-Carotene content analysis

The β-carotene content was determined by modified spectrophotometric method as previously described (Xi et al. 2020; Zhu et al. 2018a). Briefly, 1 mL of cell suspension was centrifuged at 10,000 rpm for 2 min. After centrifugation, the supernatant was discarded, and 3 mL dodecane was added. The sample was shaken vigorously to re-suspend the pellets. Then, 9 mL of methanol was added to completely break up the cells, and the tube was shaken vigorously again and then centrifuged for 2 min at 10,000 rpm. The dodecane-containing lipophilic carotenoids (upper layer) were measured with a spectrophotometer (Jasco V-530, Japan) at 453 nm and 665 nm with dodecane as reference. β-Carotene concentration was calculated using Eq. (2).

2 $${\text{C}}_{{\beta - {\text{car}}}} \left( {{\text{mg}} \cdot {\text{L}}^{{ - {1}}} } \right)\, = \,\left( {{\text{A}}_{{{453}}} - {\text{ A}}_{{{665}}} /{3}.{91}} \right) \times \,{3}.{657} \times {3} \times {\text{X}},$$

where: (A_453_ − A_665_/3.91) is the absorbance of β-carotene corrected for chlorophyll contamination, 3.657 is the calibration factor derived from HPLC analysis of β-carotene concentration, 3 is the number of milliliters of dodecane added for extraction, and X is the dilution factor to measure absorbance on spectrophotometer.

The content of β-carotene in the biomass was calculated according to Eq. (3).

3 $$\beta - {\text{carotene }}\left( \% \right)\, = \,\frac{{C_{\beta - car} \times 10}}{DW},$$

where *C*_*β-car*_ is β-carotene concentration (mg∙L^−1^), β-carotene (%) is β-carotene content, and DW is cell dry weight (mg∙L^−1^).

### Model construction methodology

Currently, the types of kinetic models, namely the Monod model and the Droop model (Zhang et al. 2015), are widely used for bioprocess simulation. Due to its high accuracy and flexibility, the Monod model is selected and modified to simulate the correlation between biomass growth and consumption of nitrate and carbon.

4 $$\frac{dX}{{dt}} = \mu_{0} *X - \mu_{d} *X^{2}$$

5 $$\mu_{0} = {\upmu }_{{{\text{max}}}} \,\left( {T,I,N,C} \right)f\left( T \right)\left( {\frac{{I_{av} }}{{I_{av} + k_{s} + \frac{{I_{av}^{2} }}{{k_{i} }}}}} \right)\left( {\frac{C}{{K_{C} + C}}} \right)\left( {\frac{N}{{K_{N} + N}}} \right)$$

6 $${\text{f}}\left( {\text{T}} \right)A*e^{{ - \frac{{E_{a} }}{R*T}}} - B*e^{{ - \frac{{E_{b} }}{R*T}}} ,$$

where *X* is biomass concentration (g·L^−1^), *µ*_*0*_ is cell-specific growth rate (h^−1^), *µ*_*max*_ is maximum cell-specific growth rate (h^−1^), *I*_*av*_ is average light intensity, *µ*_*d*_ is cell decay rate (h^−1^), *K*_*s*_ is light saturation value produced by cell growth, *K*_*i*_ is photoinhibition value of cell growth, *A* and *B* are the coefficients before the index, *E*_*a*_ is the activation energy for cell growth, *E*_*b*_ is inactivation energy of cell growth, *K*_*N*_ is the nitrate half-velocity constant, and *K*_*C*_ is carbon half-velocity constant.

Equation 4 simulates the biomass growth rate. Its first term on the right-hand side represents biomass growth, and the second term represents cell respiration and decay. In terms of β-carotene production, it was reported that the uptake of culture nitrate is essential for cells to synthesize β-carotene (Lamers et al. 2012). Meanwhile, as β-carotene is a primary carotenoid, it can be consumed by cells for their growth and converted to other metabolites when necessary. Therefore, Eq. 7 is constructed to simulate β-carotene production. In this equation, the first term on the rightepresents β-carotene synthesis rate and is originated from the Monod model, while the second term represents β-carotene consumption rate. So far, there were no reports about investigating the detailed metabolic mechanisms of β-carotene consumption.

7 $$\frac{{{\text{dw}}}}{{{\text{dt}}}} = \left[ {b + \left( {1 - \frac{{K_{NW} }}{N}} \right)} \right]*\frac{{K_{NW} }}{N}*\left( {1 - \frac{{K_{CW} }}{C}} \right)*\frac{{K_{CW} }}{C}*\left( {1 - \frac{W}{{W_{max} }}} \right)*\frac{{I_{av} }}{{I_{av} + k_{sw} + \frac{{I_{av}^{2} }}{{k_{iw} }}}}*\left[ {A_{w} *e^{{ - \frac{{E_{aw} }}{R*T}}} - B_{w} *e^{{ - \frac{{E_{bw} }}{R*T}}} } \right],$$

where *W* is β-carotene content, *b* is no correlation coefficient for the growth of β-carotene production, *K*_*sw*_ is the light saturation value of β-carotene accumulation, *E*_*aw*_ is activation energy of β-carotene accumulation; *w*_*max*_ is the maximum β-carotene content, *K*_*NW*_ is nitrate half-velocity constant for β-carotene synthesis, *I*_*av*_ is average light intensity (μmol· photons·m^−2^· s^−1^), *K*_*iw*_ is β-carotene accumulation photoinhibition value, *K*_*CW*_ is carbon content half-velocity constant for β-carotene synthesis, and *E*_*bw*_ is inactivation energy for β-carotene synthesis, the all mathematical parameters were shown in (Additional file 1: Table S1).

### Parameter estimation methodology

To obtain the best parameters of dynamic model that can fit the experimental data, the average relative error between the experimental data and the system output was used as the objective function. Moreover, the range of biomass and β-carotene concentration was used as the state constraints to establish a final nonlinear programming problem (NLP) for the parameter variables. Considering that the particle swarm optimization (PSO) is easy to be premature, in this study, we used an adaptive PSO based on sensitivity analysis to solve the problem. This algorithm can ensure that the required parameters reach the approximate global optimum, and have a certain degree of robustness. The implementation in this work is programmed in the MATLAB (R2021a) optimization environment.

## Results and discussion

### Effects of environmental factors on cell growth

To examine the effect of temperature, light intensity, and carbon and nitrate concentration on the growth kinetics of *D. salina*, different specific sets of experiments were carried out by cultivating the cells in photobioreactors (PBRs) under different temperature, average light intensity, and initial concentrations of dissolved nitrates and carbons. The experiment was set up and then monitored daily up to a total cultivation time of 96 h, when the occurrence of exponential growth was observed (Additional file 1: Fig. S2).

**Fig. 1:**
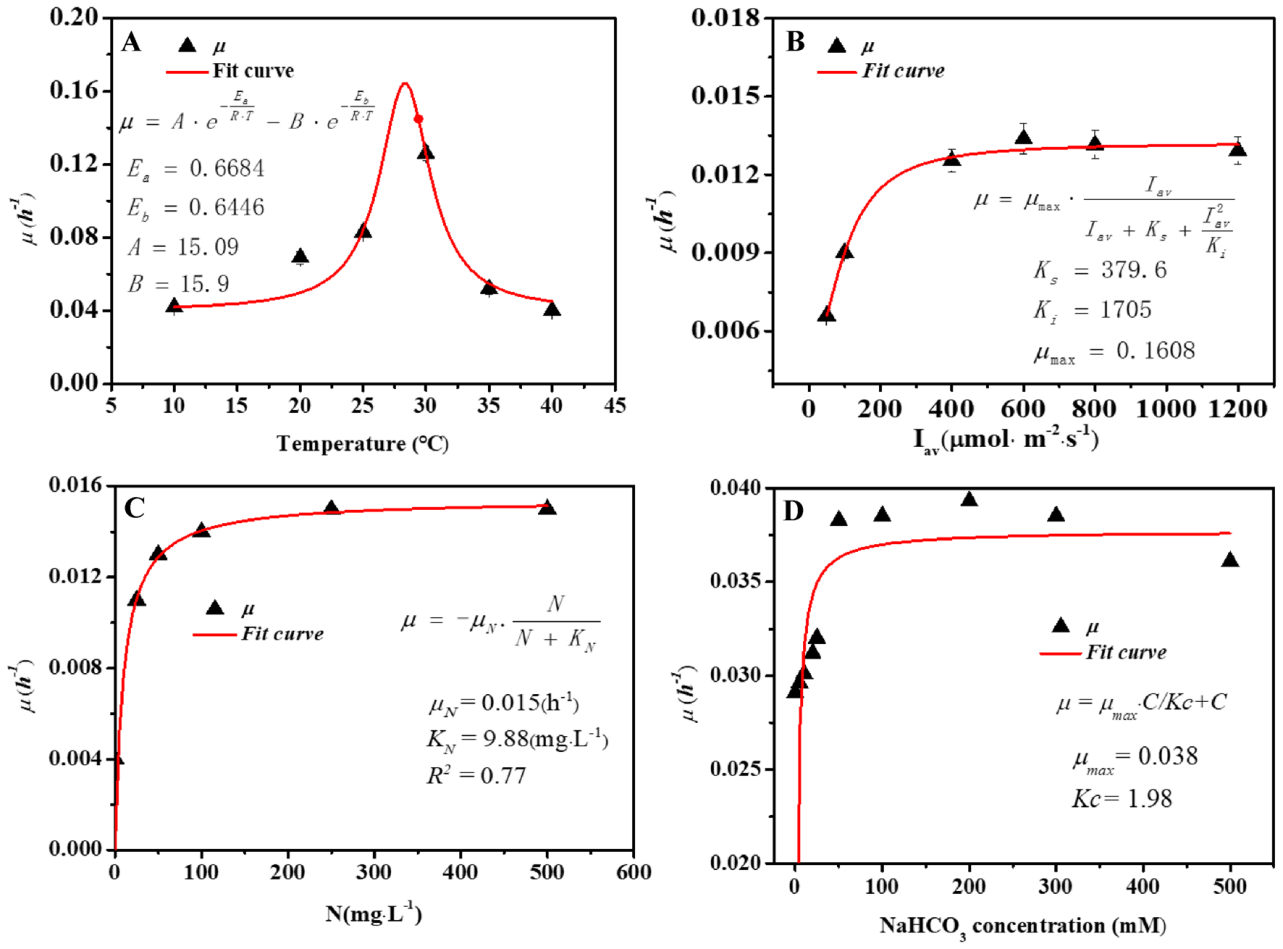
Growth rate of *D. salina* under different culture conditions. **A** The relationship of temperature and cell-specific growth rate **B** The relationship of light intensity and cell-specific growth rate. **C** The relationship of nitrogen concentration and cell-specific growth rate. **D** The relationship of carbon concentration and cell-specific growth rate

It was found that temperature remarkably influences the rate of cell growth, cell decay, and bioproduct accumulation. The optimal temperature can facilitate microbial biomass growth and bioproducts synthesis (del Rio-Chanona et al. 2017; Fachet et al. 2014; Guiheneuf and Stengel 2017). The effect of temperature on the growth rate in *D. salina* is mainly reflected in the efficiency of photosynthesis and respiration (Fachet et al. 2014). The growth rate at different temperatures is shown in Fig. 1A. At the range of 10–30 °C, the growth rate was positively correlated with temperature, and when the temperature rises to 30 °C, the growth rate begins to decrease. In the range of 30–40 °C, the growth rate has a negative correlation with temperature. The logarithmic growth phase of *D. salina* has the largest growth rate (0.164 h^−1^) at the temperature of 28 °C. The Arrhenius equation has been widely used to describe the effects of temperature on both biomass growth (Zhang et al. 2016). The parameters of the Arrhenius model can be obtained by model fitting under different range of temperatures.

Light intensity significantly affects biomass growth rates (Bonnefond et al. 2016). In general, the effect of light intensity on cell growth can be reflected by the Aiba model (del Rio-Chanona et al. 2017). In our experiments, the Aiba model was used to replace local light intensities by an average light intensity which was calculated by Eq. 5 in a suspended reactor. The model parameters *μ*_*max*_ and *K*_*s*_ were fitted, as shown in Fig. 1B, and within a certain range of light intensity, the relationship between the average light intensity and the growth rate of *D. salina* conforms to the Aiba equation. The growth rate tends to increase with the increase of light intensity stable value when the light intensity exceeds 379.6 μmol· photons·m^−2^· s^−1^. According to the curve, the half-saturation constant of light (*K*_*i*_) is 1705 μmol· photons·m^−2^· s^−1^. It was indicated that in most of the cases, a higher average light intensity (up to 600 μmol· photons·m^−2^·s^−1^) can result in a higher biomass growth rate, which was consistent with the observations published in the previous studies (Bonnefond et al. 2016; Fachet et al. 2016).

When nitrogen concentration is the limiting factor, the relationship between the growth rate and the nitrogen concentration is shown in Fig. 1C. The relationship between the nitrogen concentration and the growth rate of *D. salina* conforms to the Monod equation. The values of the model parameters *μ*_*max*_ and *K*_*N*_ after fitting show that within a certain range of nitrogen concentration, the growth rate (referred as *μ*) of cells increased with the increase of the nitrogen concentration. The *μ* tended to be a stable value when the nitrogen concentration exceeds 330 mg·L^−1^. In terms of the influence of nitrate concentration on biomass growth rate, in both sets of experiments, nitrate concentration in the culture keeps increasing after the addition of dense nitrate influent, which means that the consumption rate of nitrate due to biomass uptake was slower than its refreshment rate. By comparing biomass concentrations at the different sets of experiments, it seems that biomass growth rate was always higher in a denser nitrate concentration culture.

This tendency was also observed in the carbon experiments when the carbon content changes from 0 to 500 mM. Under the carbon limiting condition, the relationship between specific growth rate and carbon content is shown in Fig. 1D. The relationship between the carbon concentration and the growth rate conformed to the Monod equation within a certain range of carbon content in *D. salina*. The growth rate *μ* was improved as the increase of the carbon content when the carbon concentration was in a lower range. The *μ* tended to be a stable value with the increase of the carbon concentration when the carbon concentration exceeds 50 mM, above which a stable of final biomass concentration was obtained, and indicated a stable biomass growth rate. Therefore, these results suggested that the high biomass growth rate obtained with nitrate concentration 500 mg·L^−1^, carbon concentration 50 mM at 28 ℃ and the average light intensity 600 μmol· photons·m^−2^· s^−1^ (Fig. 1).

### Effects of environmental factors on β-carotene synthesis

**Fig. 2:**
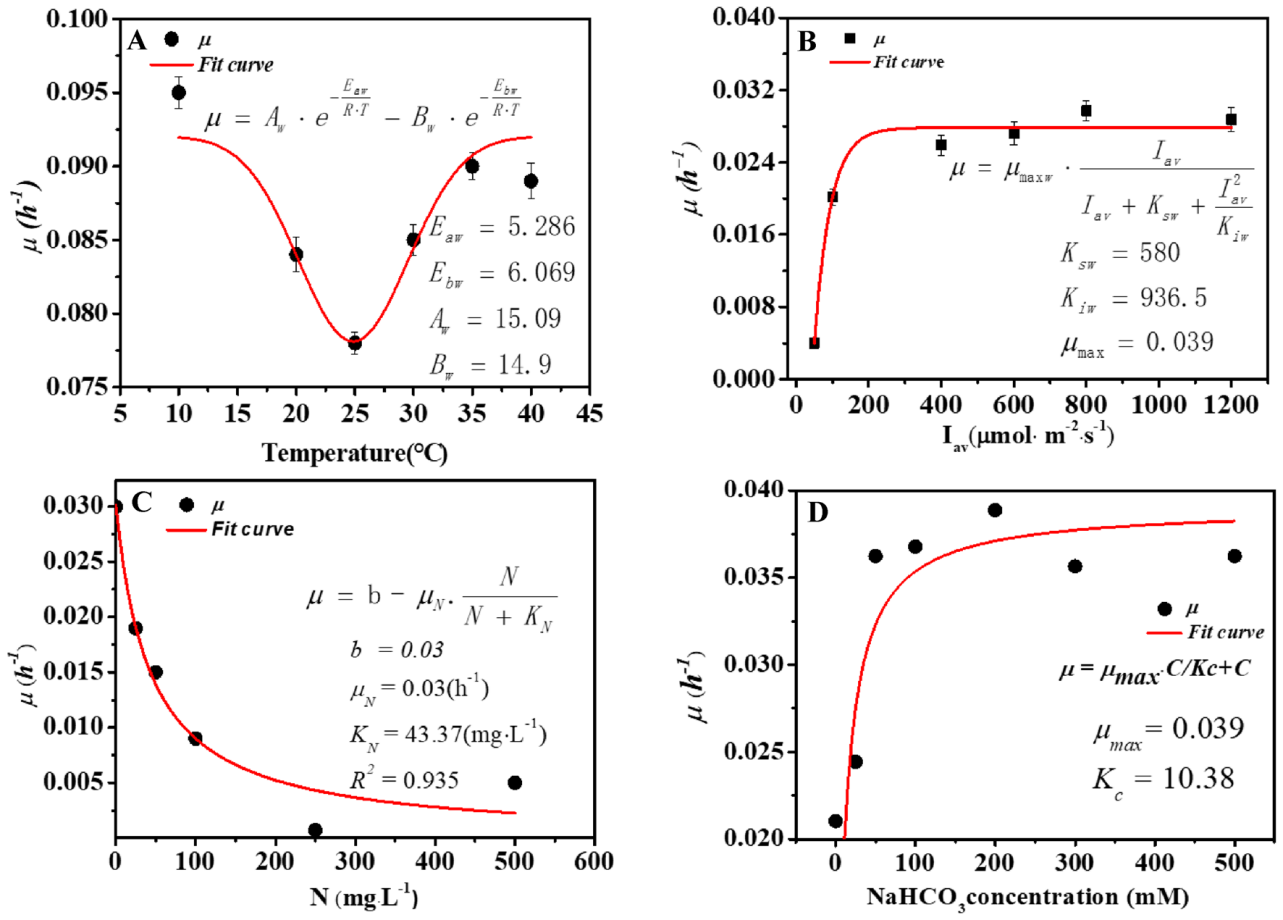
β-Carotene accumulation rate of *D. salina* under different culture conditions. **A** The relationship of temperature and β-carotene accumulation rate. **B** The relationship of light intensity and β-carotene accumulation rate. **C** The relationship of nitrogen concentration and β-carotene accumulation rate. **D** The relationship of carbon concentration and β-carotene accumulation rate

The β-carotene accumulation rate at different temperatures is shown in Fig. 2A. We found that the β-carotene accumulation rate was negatively correlated with temperature at the range of 10–35 °C, and when the temperature rises to 30 °C, the β-carotene accumulation begins to increase. In the range of 30–40 °C, the β-carotene accumulation rate has a positive correlation with temperature. The *D. salina* has the largest β-carotene accumulation rate at 10 °C, which was 0.095 h^−1^. The Arrhenius equation has been widely used to describe the effects of temperature on both bioproduct accumulation. The parameters of the Arrhenius model can be obtained by model fitting under different range of temperatures.

We found that in the experiments under different light intensities, β-carotene content continuously increases with the raising average light intensity from 150 to 600 μmol· photons·m^−2^· s^−1^, while its maximum value falls within the range of 300–480 μmol· photons·m^−2^· s^−1^ (Fig. 2B). However, neither of the current observations were in agreement with the previous studies where β-carotene content was found to decrease with the increasing light intensity from 150 to 750 μmol· photons·m^−2^· s^−1^ (Fachet et al. 2014). As a result, the distinct discrepancy between current observations and previous conclusions suggested the complex metabolic mechanisms of β-carotene synthesis. It also indicated that other factors apart from light intensity can significantly affect intracellular β-carotene content. In terms of the effect of nitrate concentration on β-carotene synthesis, it was known that nitrate is essential for β-carotene synthesis, and β-carotene was a primary carotenoid which can be accumulated under nitrogen-sufficient conditions. The results showed that under the same light intensity, β-carotene content in the experiments with lower nitrate concentration was higher than that in the experiments with higher culture nitrate concentration (Fig. 2B). This phenomenon was also reported in a recent study where a similar photosynthetic pigment lutein was synthesized by *Desmodesmus* sp. under a nitrogen-sufficient condition (del Rio-Chanona et al. 2017).

Therefore, the current observation indicated that β-carotene content was higher in a lower nitrate concentration condition which could also be explained as what has been demonstrated for β-carotene. Under the carbon deprivation condition, the relationship between β-carotene accumulation and carbon concentration is shown in Fig. 2D, and within a certain range of carbon concentration, the relationship between the carbon concentration and the specific accumulation rate conformed to the Monod equation in *D. salina*. The β-carotene accumulation rate in microalgae increased with the higher concentration of the carbon. The *μ* tended to be a stable value with the increase of the carbon concentration when the carbon concentration exceeds 200 mM.

For β-carotene production, the results showed that a higher light intensity and a denser culture nitrate concentration frequently led to a higher β-carotene production as long as nitrate inhibition does not happen, as shown in Fig. 2B. Such conflicting conclusion compared to that of β-carotene synthesis was reasonable, since β-carotene production is the product of both biomass concentration and β-carotene intracellular content. Although a high nitrate concentration may limit β-carotene accumulation, it can significantly facilitate green microalgae biomass growth. Consequently, total β-carotene production can still be increased through this condition. Nonetheless, it should be noted that low β-carotene content can remarkably elevate the bioprocess downstream separation cost, which may seriously reduce the process profitability. Hence, it is essential to guarantee an adequate β-carotene content when aiming to maximize total β-carotene production.

### Results of dynamic model construction

To construct a highly accurate dynamic model which is capable of simulating the performance of green microalgal β-carotene production, and accomplish further process optimization, it is vital to understand the biochemical kinetics of the investigated system. Especially for the current process, the temperature, light intensity, carbon content, and culture nitrate concentration should be included in the model, since previous studies have declared that they are the main factors affecting β-carotene synthesis (Bonnefond et al. 2016; Lamers et al. 2012). From the “Growth analyses” section and “β-carotene content analysis” section, parameters in the kinetic model were calculated through single factor experiments, as shown in Table 1. We found that the specific biomass decay rate was not equal 0, which indicated that they have not negligible effects on the system. This can be attributed to the fact that in all the conducted experiments, biomass concentration kept steady until the end of the study, and the effect of cell decay should not disguise. The parameters were used as the initial value of the parameter for further parameter identification. Considering that a single perturbation method may affect the accuracy of the results, to reduce the sensitivity of the parameters from the perturbation method, we randomly perturb each parameter Q times and draw a box plot of the objective function with respect to the perturbation percentage.

**Table 1:** Parameters based on average light intensity

Parameter	Unit	Value
K_s_	μmol· photons·m^−2^· s^−1^	379.6
E_a_	kJ mol^−1^	0.6684
E_b_	kJ mol^−1^	0.6449
E_aw_	kJ mol^−1^	5.286
E_bw_	kJ mol^−1^	6.069
K_sw_	μmol· photons·m^−2^· s^−1^	580
A		15.09
B		14.9
A_w_		0.4355
B_w_		0.35
µ_max_	h^−1^	0.1608
w_max_	h^−1^	0.039
K_i_	μmol· photons·m^−2^· s^−1^	1705
K_iw_	μmol· photons·m^−2^· s^−1^	936.5
K_N_		9.88
K_C_		0.22
K_CW_		10.38
K_Nw_		43.37
b		0.03
μ_d_	h^−1^	0.3

**Fig. 3:**
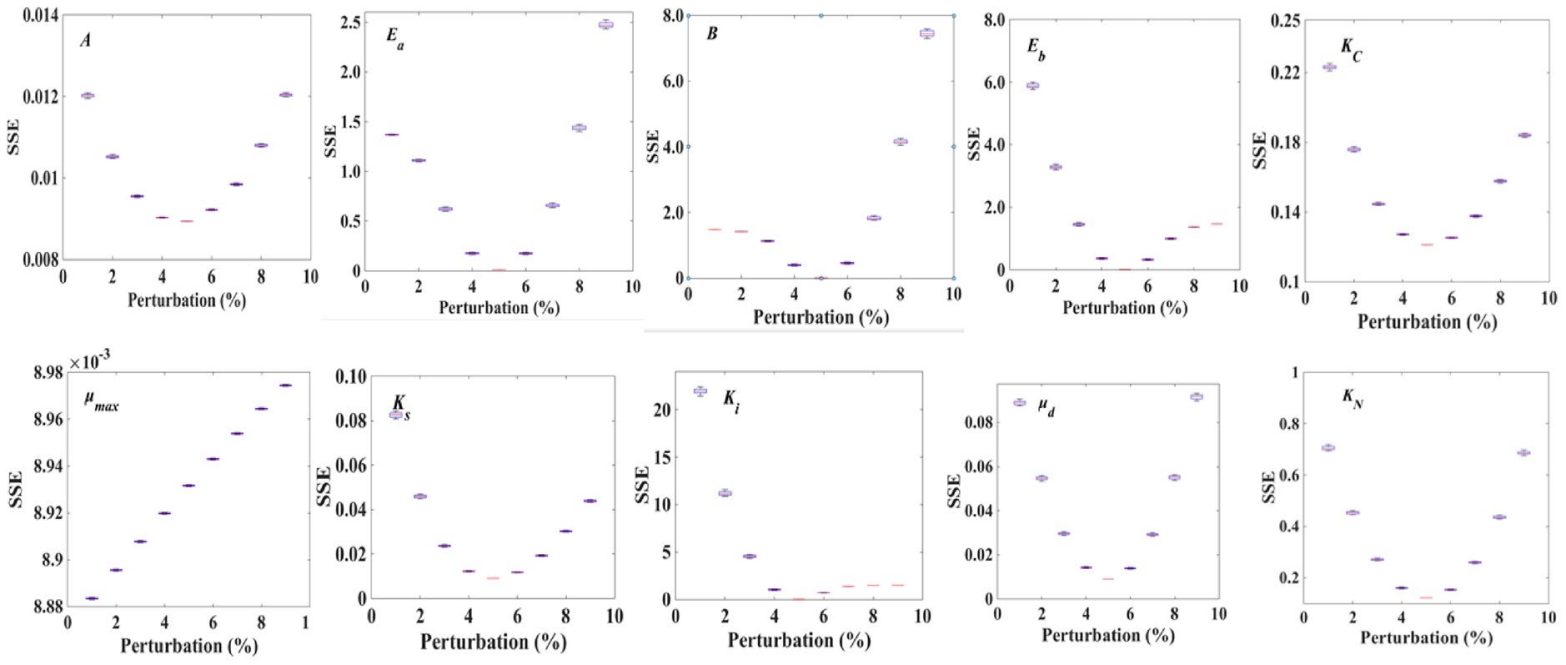
Optimization of model parameters for cell growth in *D. salina* (optimized by MATLAB). SSE: system squared error. Where *A* and *B* are the coefficients before the index, *E*_*a*_ is the activation energy for cell growth, *E*_*b*_ is inactivation energy of cell growth, *K*_*C*_ is carbon half-velocity constant, µ_max_ is maximum cell-specific growth rate, *Ks* is light saturation value produced by cell growth, *K*_*i*_ is photoinhibition value of cell growth, *K*_*N*_ is the nitrate half-velocity constant, *µ*_*d*_ is cell decay rate

**Fig. 4:**
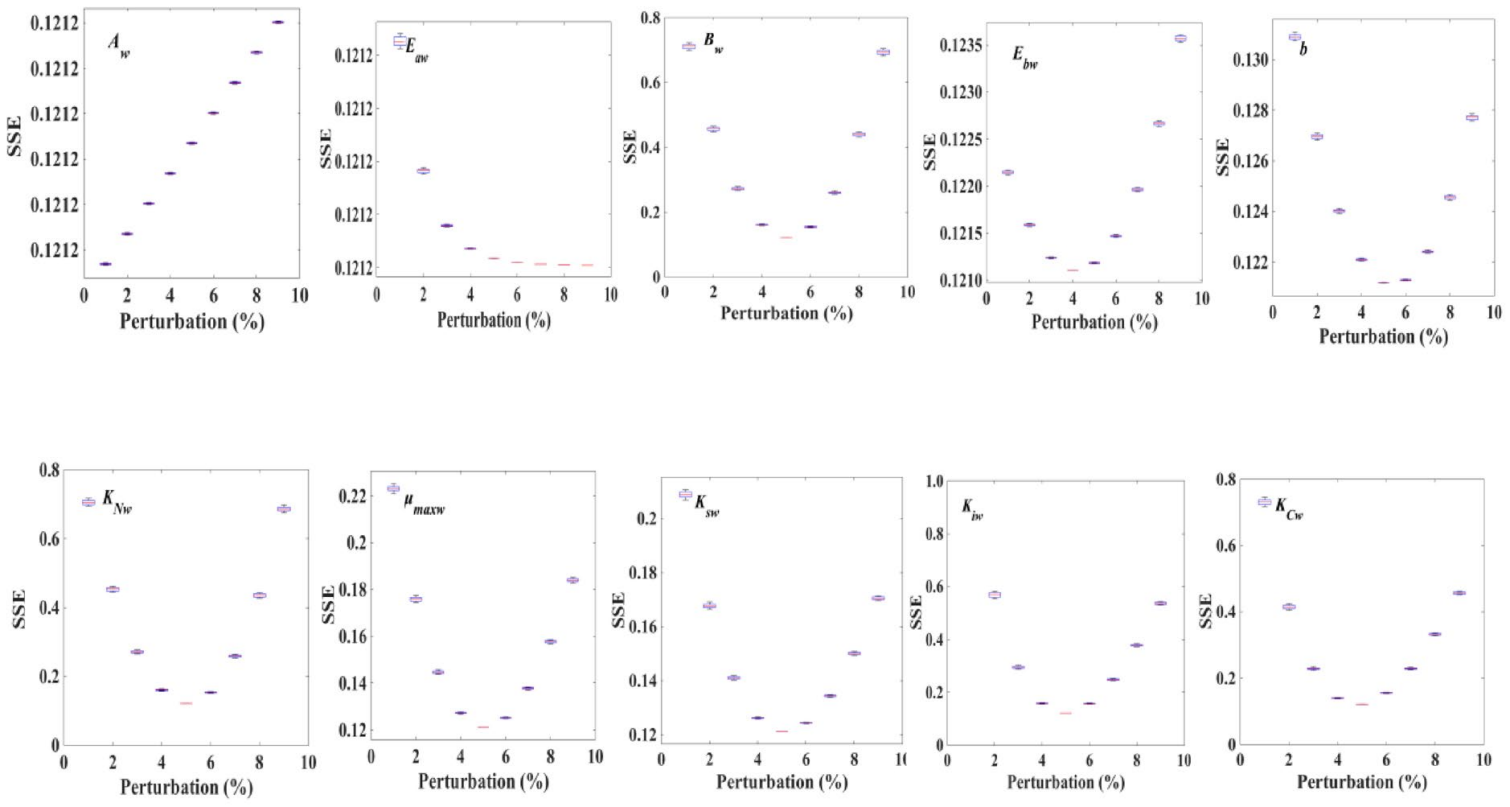
Optimization of model parameters for β-carotene accumulation in *D. salina* (optimized by MATLAB). SSE: system squared error, where *A*_*w*_ and *B*_*w*_ are the coefficients before the index, *E*_*aw*_ is activation energy of β-carotene accumulation, *E*_*bw*_ is inactivation energy for β-carotene synthesis, *b* is no correlation coefficient for the growth of β-carotene production, *K*_*sw*_ is the light saturation value of β-carotene accumulation, *μ*_*wmax*_ is the maximum β-carotene content, *K*_*Nw*_ is nitrate half-velocity constant for β-carotene synthesis, *I*_*av*_ is average light intensity, *K*_*iw*_ is β-carotene accumulation photoinhibition value, and *K*_*Cw*_ is carbon content half-velocity constant for β-carotene synthesis

In the process of parameter simulation, the disturbances time Q = 100, disturbance range ζ = 5%, and the optimal parameters value of the system were obtained with the aid of the PSO algorithm. The optimized parameters resulted in a box plot of the objective function change under a certain perturbation percentage, as shown in Table 2, Figs. 3 and 4, in which *E*_*b*_, *A*_*w*_, and *b* obtain the minimum value of the objective function within the perturbation range of the corresponding parameter ± 5%, and *E*_*a*_ decreases monotonically within the perturbation range of the parameter. However, within this range, the maximum difference of the objective function value is 0.016 × 10^–3^. It was suggested that the system is not sensitive to changing the parameters, and the system reaches the approximate global optimal solution of the objective function at 0 perturbation of the parameters. These results showed that the parameters obtained by the above algorithm can fit the experimental data well, and the parameters created in this study were an approximate global optimal solution of the nonlinear dynamic system and have certain robustness.

**Table 2:** Optimization of model parameters (optimized by MATLAB)

Parameter	Unit	Value (prior to Opt)	Value (after Opt)
K_s_	μmol· photons·m^−2^· s^−1^	379.6	384.9
E_a_	kJ mol^−1^	0.6684	1.0026
E_b_	kJ mol^−1^	0.6449	0.5572
E_aw_	kJ mol^−1^	5.286	7.9290
E_bw_	kJ mol^−1^	6.069	3.0345
K_sw_	μmol· photons·m^−2^· s^−1^	580	581.6
A		15.09	22.6350
B		14.9	17.4630
A_w_		0.4355	0.6533
B_w_		0.35	0.1750
µ_max_	h^−1^	0.1608	0.20278
w_max_	h^−1^	0.039	0.0405
K_i_	μmol· photons·m^−2^· s^−1^	1705	1707
K_iw_	μmol· photons·m^−2^· s^−1^	936.5	937.1
K_N_		9.88	10.23
K_C_		0.22	0.26
K_CW_		10.38	10.5
K_NW_		43.37	45.98
b		0.03	0.0150
μ_d_	h^−1^	0.3	0.15

Opt stands for optimization

### Validation of dynamic model predictability

To estimate the optimal operating conditions for long-term bioprocess optimization, besides accurately representing a known experiment, the model should possess great predictive capability when simulating unknown processes. For this reason, the predictive capability of the constructed model was investigated through two scenarios. In the first scenario, the model was used to predict the dynamic performance of a continuous illumination batch experiment lasting for 6 day indoors. In the second scenario, the model was applied to predict a light/dark cycle batch experiment lasting for 6 days under outdoor condition. Noticeably, due to the frequent change of light intensity, the second system becomes more complex and has a higher uncertainty compared to the first scenario. Both light intensity and initial nitrate concentration in these two experiments are different from those used for model construction. The detailed operating conditions of these experiments are listed in Table 3.

**Table 3:** List of arbitrary cultivation condition parameter value

Cultivation conditions	Initial DW (g·L^−1^)	Day/night (h:h)	Temperature (℃)	Light (μmol· photons·m^−2^· s^−1^)	Carbon (mM)	Nitrogen (mg·L^−1^)
A	0.1	14:10	20	200	5	50
B	0.1	24:0	25	600	5	500
C	0.1	24:0	20	800	200	50
D	0.1	14:10	25	1000	200	500

**Fig. 5:**
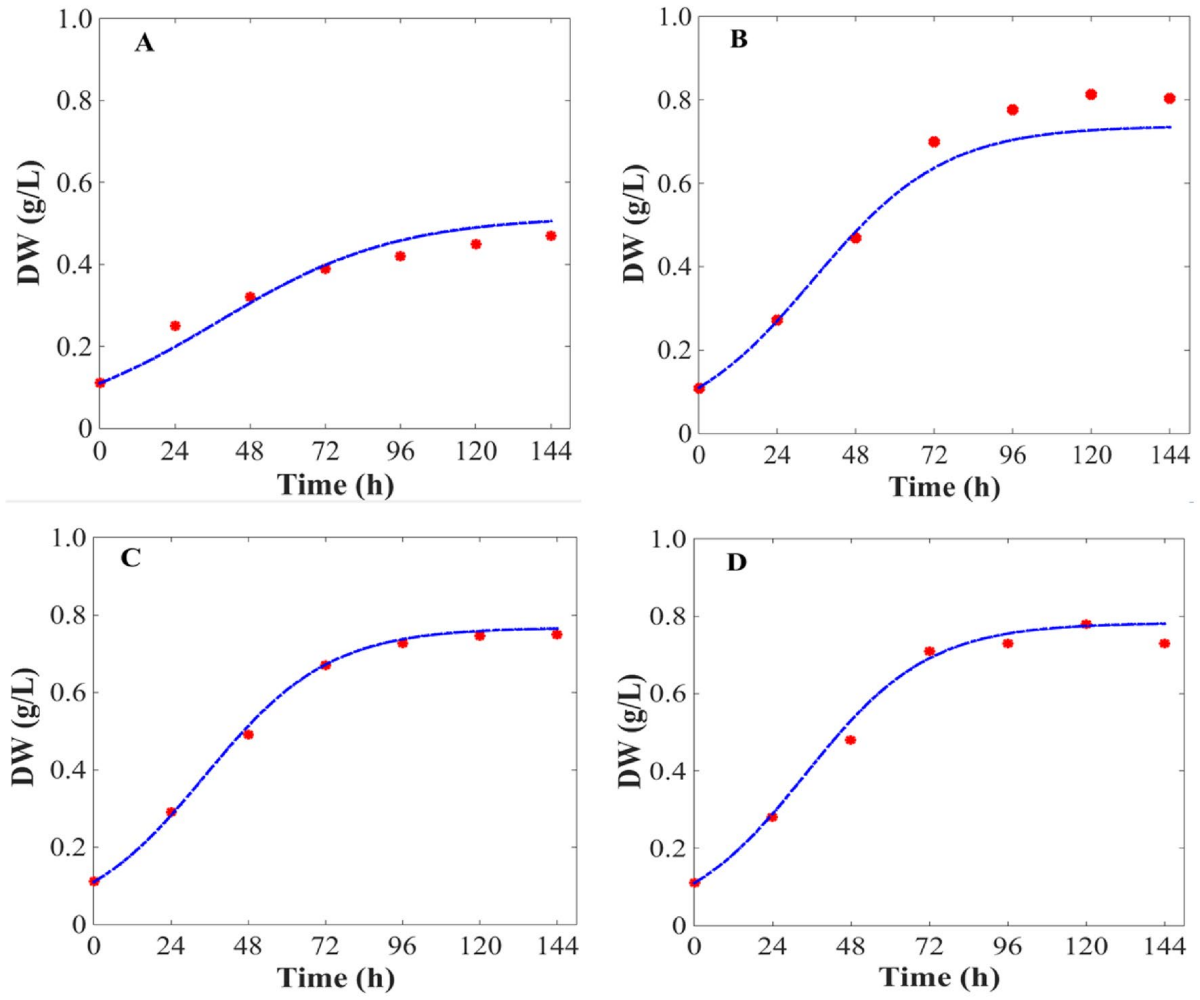
Comparison of model simulation results and real experimental data of biomass in *D. salina*. **A–D** were the arbitrary cultivation condition parameter values showed in Table 3, and the initial DW was 0.1 g·L^−1^; Day/Night were 14 h:10 h, 24 h:0 h, 24 h:0 h, and 14 h:10 h; the temperature was 20, 25, 20, and 25℃, light intensity were 200, 600, 800, and 1000 μmol· photons·m^−2^· s^−1^; the carbon concentrations were 5, 5, 200, and 200 mM; and the nitrogen concentration was 50, 500, 50, and 500 mg·L^−1^, respectively. The Lines indicate simulation results, and the points indicate experimental measurements. DW, dry weight

**Fig. 6:**
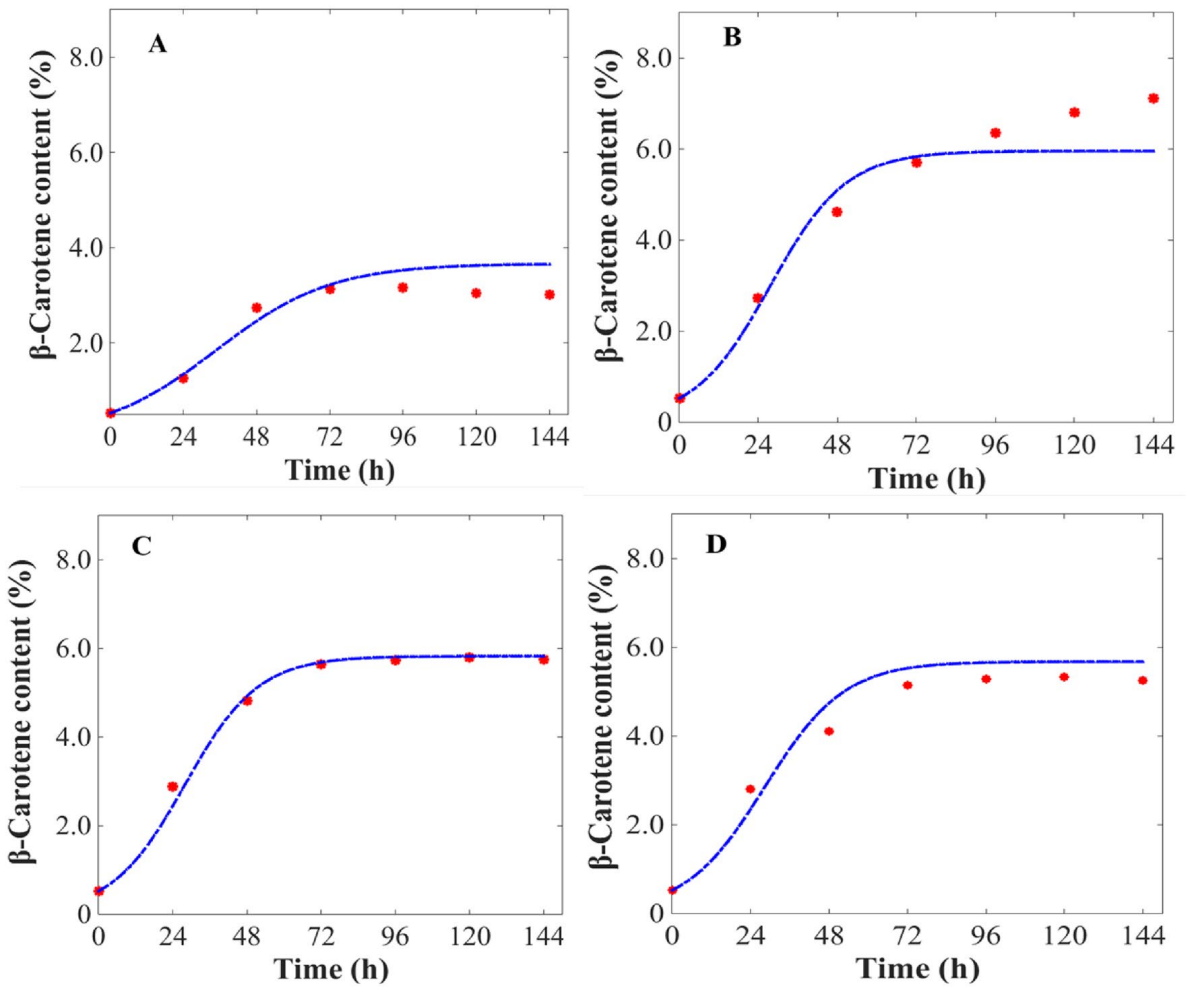
Comparison of model simulation results and real experimental data of β-carotene content in *D. salina*. **A–D** were the arbitrary cultivation condition parameter values showed in Table 3, and the initial dry weight was 0.1 g·L^−1^; Day/Night were 14 h:10 h, 24 h:0 h, 24 h:0 h, and 14 h:10 h; the temperature was 20, 25, 20, and 25℃, light intensities were 200, 600, 800, and 1000 μmol· photons·m^−2^· s^−1^; the carbon concentrations were 5, 5, 200, and 200 mM; and the nitrogen concentration was 50, 500, 50, and 500 mg·L^−1^, respectively. The lines indicate simulation results and the points indicate experimental measurements

To identify the predictability of current models for β-carotene production process in different *D. salina* strains, four additional experiments were carried out. The experiments have the initial biomass concentration of 0.10 g·L^−1^, with incident light intensity of 200, 600, 800, and 1000 μmol· photons·m^−2^·s^−1^ and temperature of 20 and 25 °C. The model was validated by Algal station system with 1.0 L PBR (Additional file 1: Fig. S1), steady temperature, and light intensity within the reactor operating conditions. Despite the added variability, the model was able to accurately reproduce the process performance with the same calibrated values for all the model parameters. The comparison between the current model simulation results and experimental data was examined. It was shown that the current models can accurately predict the dynamic performance of green microalgal β-carotene production process with different operating conditions (Figs. 5 and 6). Because the current dynamic model was constructed with the aim to predict the optimal operating conditions in future process design and control, it has to be characterized by not only high accuracy but also good predictability. Therefore, this model is used to simulate the dynamic performance of all the remaining four experiments conducted in this study. We found that the current model showed great predictability within a wide range of operating conditions throughout the entire experimental time-course. Among the all the experimental data points, the majority of deviation between model prediction and real experiment was far below 10%, with only four exceptions shown in Fig. 5A (11.4%) and Fig. 6C (13.8%). Therefore, it was strongly indicated the current model showed high predictability and accuracy of the current model, as well as its great competence for further process design and optimal control.

Our model was fitted with experimental data that accounted for the most significant parameters that affect *D. salina* metabolism, which was intended to promote and assist the development of evaluation applications of *D. salina* at industrial level. Moreover, our model could also be used as a predictive tool to determine the combination of environmental and operational parameters that promote maximum biomass productivity and bioproduct production in other microalgae. Methods developed in this study might be helpful for establishing a model to simulate β-carotene production in other microalgae species.

## Conclusions

In this study, a mathematical model was constructed to simulate the growth and β-carotene production from *D. salina*. Sensitivity analysis showed that β-carotene synthesis is more sensitive to the operating parameters of the system than cell growth. Moreover, the accuracy and predictability of kinetic model were further verified. Based on the dynamic model, optimal light intensities for cell growth and β-carotene production were proposed. The established model has high accuracy and predictive capability, which is potentially useful for further application in process control and optimization during microalgae cultivation at industrial level.

### Supplementary Information

**Additional file 1:** **Figure S1.** The schematic overview of the Algal Station platform and light and temperature automatic control platform adapted from Cao et al. 2019. **Figure S2.** The time-course microscopic images of the *D.* *salina* green cells turning into yellowish or orange under light condition of 800 μmol· photons·m−2·s−1. **Table S1.** Mathematical parameters

## Data Availability

All data generated or analyzed during this study are included in this article.
