# Supplementary material for: Kinetic modeling and process analysis for photo-production of β-carotene in Dunaliella salina
Source: Bioresour Bioprocess. 2022 Jan 17;9(1):4. doi: 10.1186/s40643-022-00495-6 (PMC10991233; doi:10.1186/s40643-022-00495-6)
Supplement: Supplementary file 1 — Additional file 1: Figure S1. The schematic overview of the Algal Station platform and light and temperature automatic control platform adapted from Cao et al. 2019. Figure S2. The time-course microscopic images of the D. salina green cells turning into yellowish or orange under light condition of 800 μmol· photons·m−2·s−1. Table S1. Mathematical parameters [file 40643_2022_495_MOESM1_ESM.docx]

**Supplementary Figures and Tables**


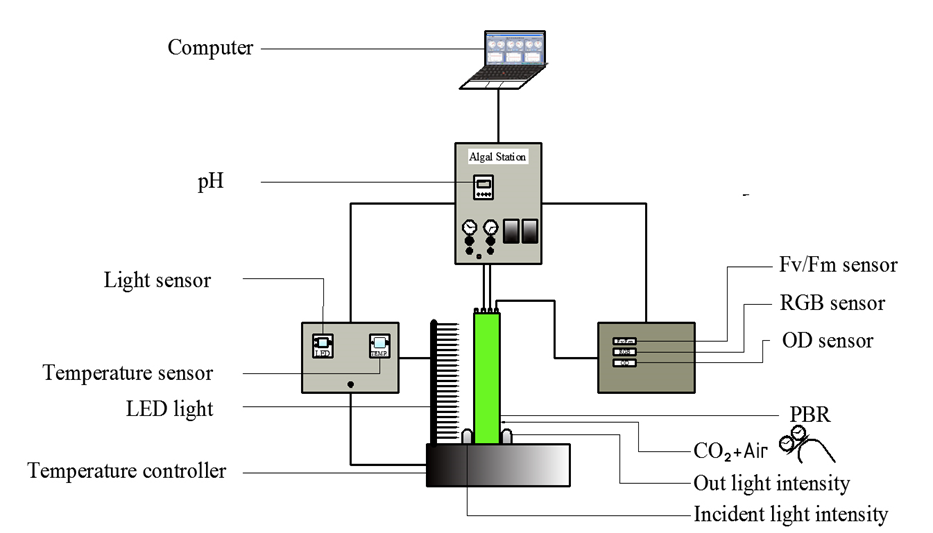


**Fig. S1**. The schematic overview of the Algal Station platform and light and temperature automatic control platform adapted from Cao et al., 2019.


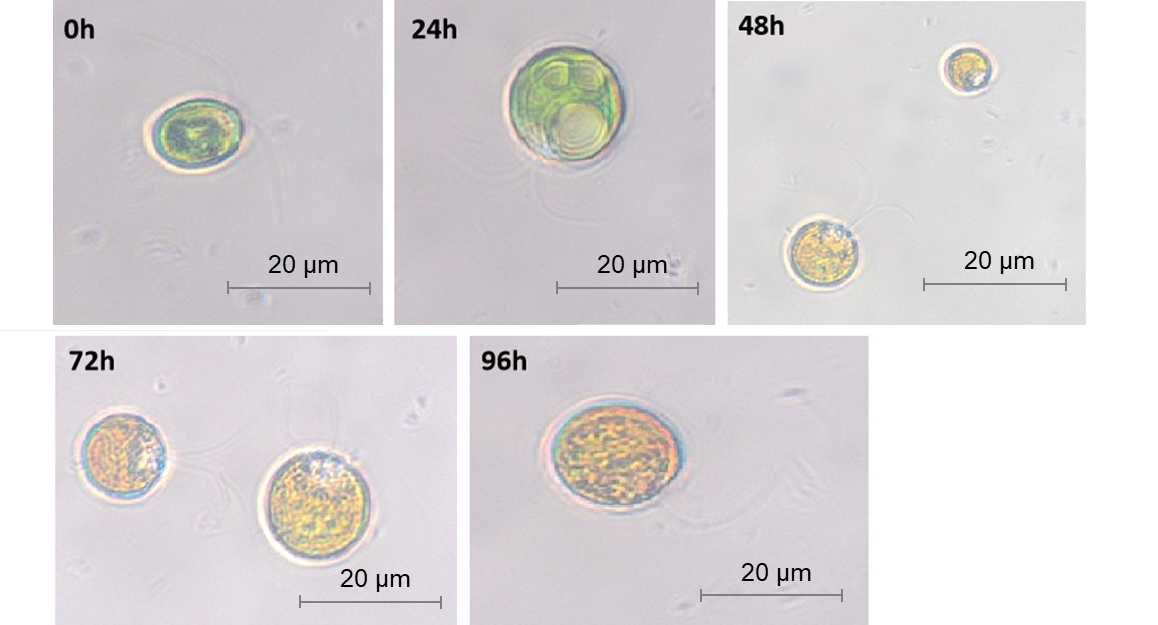


**Fig. S2**. The time-course microscopic images of the *D. salina* green cells turning into yellowish or orange under light condition of 800 μmol· photons·m^−2^· s^−1^.

**Table S1. Mathematical parameters**

| Parameter | Value | Unit |
| --- | --- | --- |
| K_s_ | The light saturation value produced by cell growth | μmol· photons·m^−2^· s^−1^ |
| E_a_ | The activation energy for cell growth | kJ mol^−1^ |
| E_b_ | The inactivation energy of cell growth | kJ mol^−1^ |
| E_aw_ | The activation energy of β-carotene accumulation | kJ mol^−1^ |
| E_bw_ | The inactivation energy for β-carotene synthesis | kJ mol^−1^ |
| K_sw_ | The light saturation value of β-carotene accumulation | μmol· photons·m^−2^· s^−1^ |
| A | The coefficients before the index |  |
| B | The coefficients before the index |  |
| A_w_ | The coefficients before the index |  |
| B_w_ | The coefficients before the index |  |
| µ_max_ | The maximum cell specific growth rate | h^-1^ |
| w_max_ | The maximum β-carotene content | h^-1^ |
| K_i_ | The photoinhibition value of cell growth | μmol· photons·m^−2^· s^−1^ |
| K_iw_ | The β-carotene accumulation photoinhibition value | μmol· photons·m^−2^· s^−1^ |
| K_N_ | The nitrate half-velocity constant |  |
| K_C_ | The carbon half-velocity constant |  |
| K_CW_ | The carbon content half velocity constant for β-carotene synthesis |  |
| K_Nw_ | The nitrate half velocity constant for β-carotene synthesis |  |
| b | There is no significant correlation coefficient for the growth of β-carotene production |  |
| μ_d_ | The cell decay rate | h^-1^ |
